# The ability to drive cortical networks in the low gamma range differs between sexes

**DOI:** 10.1016/j.isci.2026.114893

**Published:** 2026-02-04

**Authors:** Aurimas Mockevičius, Inga Griškova-Bulanova

**Affiliations:** 1 Vilnius University, Institute of Bioscience, Life Sciences Center, Saulėtekio Ave. 7, Vilnius 10257, Lithuania; 2 Vilnius University, Translational Health Research Institute, Faculty of Medicine, Žaliųjų ež. str. 2, Vilnius 08406, Lithuania

**Keywords:** Neuroscience, Sensory neuroscience, Cognitive neuroscience

## Abstract

The brain’s ability to synchronize with periodic auditory stimuli is widely used to study gamma-range neural activity. Chirp-like stimulation, covering a broad frequency range, elicits envelope-following responses (ERFs) that enable assessment across multiple frequencies and estimation of the individual gamma frequency (IGF). However, the impact of sex differences on EFR remains understudied. We compared auditory ERFs in the 30–60 Hz range between females and males. Electroencephalography was recorded in 80 healthy young adults (42 females; 26.07 ± 4.28 years) using chirp-like auditory stimulation. Females were tested during the early follicular phase to minimize hormonal effects. Phase-locking index (PLI) and event-related spectral perturbation (ERSP) were analyzed across nine fronto-central electrodes. Females showed significantly lower PLI (35–43 Hz) and ERSP (35–46 Hz), while IGFs were comparable. These results emphasize sex-related influences on gamma auditory responses and the need for sex-specific normative baselines in clinical applications.

## Highlights

• Auditory chirp-like click stimulus covering 30–60 Hz elicits frontocentral gamma EFR
• Stronger and more synchronized 35–45 Hz EFR in males compared to females
• Individual gamma frequency derived from EFRs did not differ between sexes
• No topographic lateralization observed in gamma EFRs

## Graphical abstract

**Figure:**
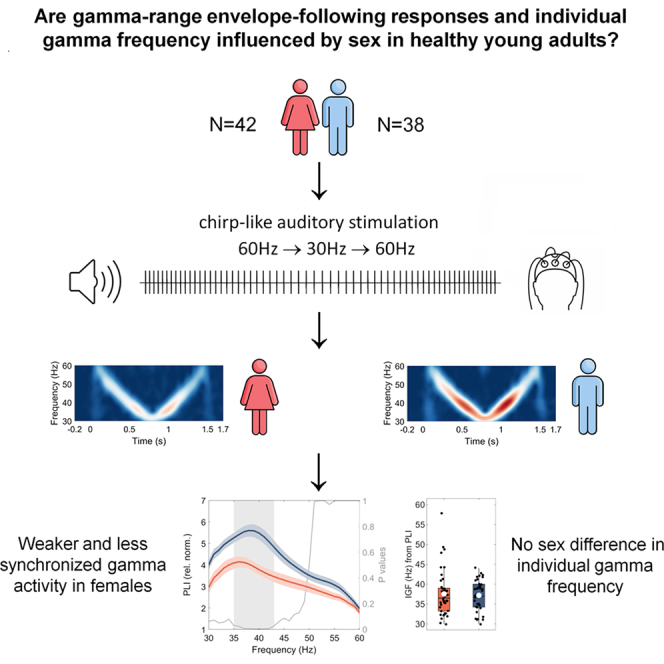

## Introduction

Neural oscillations represent synchronized patterns of brain activity which play a pivotal role in the transmission and integration of information across brain networks.^1^ Specifically, oscillatory activity in the gamma frequency range (>30 Hz) reflect bottom-up sensory and cognitive information processing.^1^^,^^2^ Given their importance, methods aiming to reliably assess synchronized gamma oscillations are being investigated, which include auditory steady-state responses (ASSR).^3^ ASSR is a continuous synchronized activity entrained by rhythmic auditory stimulus. This response is characterized by stability in amplitude and phase and is frequently used to examine neural dynamics in various neuropsychiatric disorders^4^ and associated cognitive impairments^5^; even more, it has been proposed as a potential biomarker for several conditions, including schizophrenia, bipolar disorder, and autism.^6^^,^^7^^,^^8^ With the growing number of studies employing this paradigm, it is becoming increasingly important to establish how brain responses to periodic auditory stimulation relate to individual state and trait characteristics for accurate and objective interpretation.

Sex is an important biological trait that determines both structural and functional differences at multiple levels of organization within the body, including the brain.^9^ A growing body of evidence suggests that sex plays a crucial role in the prevalence, symptomatology, and course of psychiatric disorders.^10^ For instance, schizophrenia tends to manifest earlier and with greater severity in males, whereas females experience a later onset with a relatively milder course.^11^^,^^12^ Similarly, in bipolar disorder, females exhibit higher rates of rapid cycling and anxiety, while males present a greater number of hospitalizations for mania and a higher likelihood of comorbid substance use.^13^^,^^14^ Moreover, autism spectrum disorders are often underdiagnosed in females due to sex-related differences in symptom presentation, with females more likely to mask symptoms and display internalizing behaviors such as anxiety.^15^^,^^16^ These observations underscore the necessity of incorporating sex as a critical factor in the development of diagnostic frameworks and treatment strategies, including research aimed at identifying potential biomarkers of brain function. Despite this, to our knowledge, the influence of sex on gamma-range ASSRs has not been systematically investigated.

The limited existing research in both humans and animal models suggests that sex and sex hormones modulate ASSR, emphasizing the importance of sex-specific normative baselines: (1) females exhibit lower ASSR thresholds than males, particularly at low carrier frequencies^17^; (2) sex differences have been reported in small samples of left-handed individuals, with females showing weaker 40-Hz ASSRs than males, whereas right-handed individuals did not exhibit such differences^18^; (3) animal study indicated stronger 40-Hz ASSRs in male mice when compared to female mice^19^; but (4) in a mixed sample of schizophrenia patients and controls, stronger 40-Hz ASSR amplitude was observed in females.^20^ Importantly, in females (both human and animal models), fluctuations in estrogen levels have been shown to cause significant variations in the magnitude of the 40-Hz ASSR.^21^^,^^22^ These observations, together with documented sex-related differences in auditory processing at subcortical^23^^,^^24^ and cortical levels^25^^,^^26^ suggest the presence of inherent sex-related differences in gamma-band synchronization, which could stem from differences in neural circuitry and/or hormonal modulation.

Notably, most existing studies used ASSR paradigm and focused on a single frequency—40 Hz. Alternatively, envelope-following responses (EFRs) across a broad range of input frequencies can be efficiently assessed using a chirp-like stimulus, which linearly^27^ or logarithmically^28^ sweeps through range of frequencies over time. This method enables the rapid characterization of frequency-specific responses, providing a more comprehensive assessment of gamma-range synchronization that was proven useful in various contexts.^29^^,^^30^^,^^31^^,^^32^ Additionally, this approach enables efficient estimation of the individual resonance frequency within the gamma range, referred to as the individual gamma frequency (IGF), which represents the frequency at which the strongest neural response is observed during stimulation.^33^ IGF is thought to reflect individual differences in network properties,^34^ which are shaped by anatomical structure and the speed of neuronal communication^35^—both of which are known to differ between sexes.^36^^,^^37^ Despite these theoretical implications, sex differences in auditory IGF have not been systematically explored. Research suggests that individual variation in gamma frequency is functionally relevant,^38^ influencing auditory processing in both healthy individuals (e.g., gap detection sensitivity^39^^,^^40^ and in neuropsychiatric conditions (e.g., symptom severity^41^^,^^42^). Given the increasing importance of gamma-range neural synchronization assessment in both basic and clinical research, understanding sex differences in these responses is of outmost importance for refining diagnostic and therapeutic applications.

**Figure 1:**
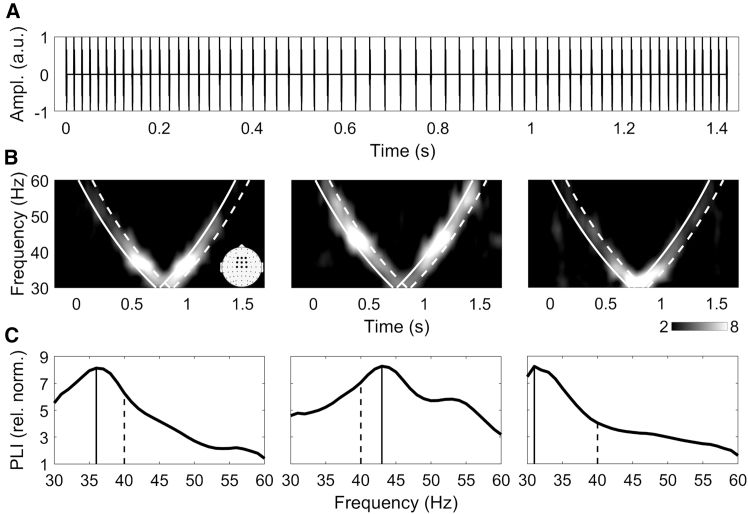
Auditory stimulation and EFR analysis (A) Auditory stimulus representation. (B) Time-frequency plots of PLI responses to the auditory stimulus of three individual subjects. Solid line represents the stimulus time, dashed line depicts the edge of the time window (+150 ms relative to stimulus time) in which PLI values were averaged. Topography shows the frontocentral electrodes which were averaged to measure the response. (C) PLI values averaged in the time window and the frontocentral channels of the corresponding subjects. Solid line represents the frequency with the highest PLI (IGF), dashed line marks PLI at 40 Hz. For ERSP, the procedures were identical.

Leveraging the EFR approach, the present study aimed to investigate sex differences in gamma-range activity by examining phase-locking properties, response strength, and IGF using low-gamma auditory click-based chirp-like stimulation (see Figure 1 for the visualisation of methods) in a homogenous sample of healthy young male and female participants.

## Results

Group characteristics and comparisons are provided in Table 1. Female and male samples did not differ in terms of age, education level and experienced symptoms of depression and anxiety. Age, sex, and race of each participant is provided in Supplementary materials (Table S1).

**Table 1:** Group characteristics and their statistical comparisons using Wilcoxon Rank-Sum test (Age, BAI, BDI) and Chi-Squared test (Education)

	N	Age (M ± SD)	Education (% tertiary)	BAI (M ± SD)	BDI (M ± SD)
Females	42	25.48 ± 4.05	79%	30.83 ± 6.49	9.93 ± 8.01
Males	38	26.97 ± 4.31	68%	28.89 ± 6.67	8.05 ± 6.40
		z = −1.52, *p* = 0.13	χ^2^ = 1.06, *p* = 0.3	z = 1.64, *p* = 0.1	z = 0.81, *p* = 0.42

BAI, Beck Anxiety inventory; BDI, Beck Depression Inventory.

**Figure 2:**
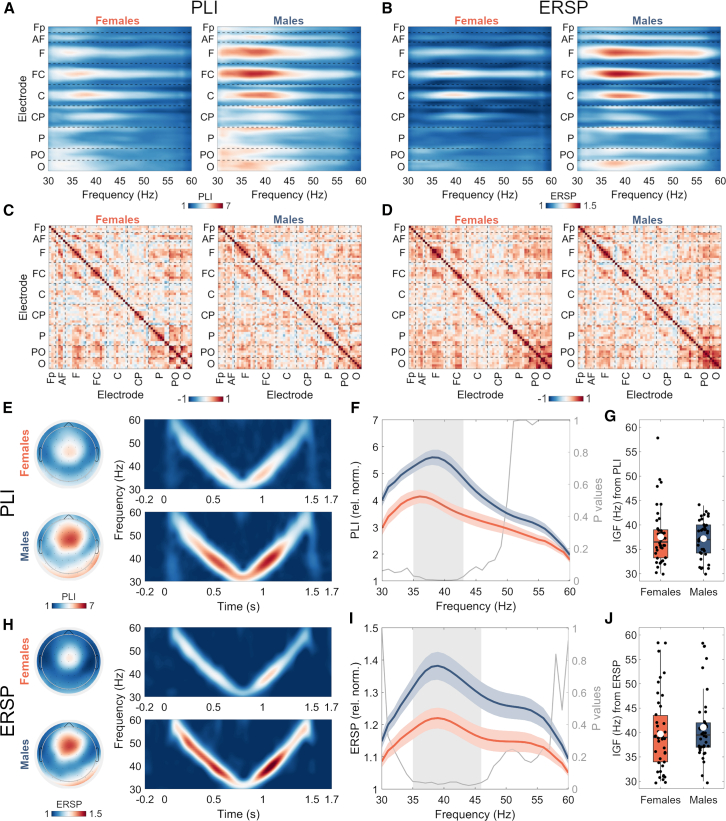
Auditory envelope-following responses in females and males First row: grand-averaged single-electrode PLI (A) and ERSP (B) frequency curves (30–60 Hz). Second row: Spearman correlation coefficients for single-electrode-based IGFs extracted from PLIs (C) and ERSPs (D). For visualization purposes, electrodes are clustered into ROIs arranged on the anterior-posterior plane, and separated by dashed lines for clarity. Fronto-polar/Fp (Fp1, Fpz, and Fp2); anterio-frontal/AF: AF7, AF3, AF4, and AF8); frontal/F (F7, F5, F3, F1, Fz, F2, F4, F6, and F8); fronto-central/FC (FT7, FC5, FC3, FC1, FCz, FC2, FC4, FC6, and FT8); central/C (T7, C5, C3, C1, Cz, C2, C4, C6, and T8); centro-parietal/CP (TP7, CP5, CP3, CP1, CPz, CP2, CP4, CP6, and TP8); parietal/P: (P7, P5, P3, P1, Pz, P2, P4, P6, and P8); parieto-occipital/PO (PO5, PO3, POz, PO4, and PO6); occipital/O: (PO7, O1, Oz, O2, and PO8). On the *y* axis of the plots, left-sided electrodes are depicted below the midline and right-sided electrodes are placed above the midline of each ROI. Third and fourth row: Topographies and time-frequency plots of PLI (E) and ERSP (H) in female and male groups are shown on the left. Topographies represent the average responses over the frequencies showing statistically significant differences (F and I) and time-frequency plots depict responses to chirp stimulus in both groups, averaged over fronto-central electrodes (F1, Fz, F2, FC1, FCz, FC2, C1, Cz, and C2). On the right, grand-averaged frequency curves and extracted IGF distributions of PLI (F and G) and ERSP (I and J) in females (orange) and males (blue). In EFR curves, transparent area indicates standard errors of the mean (SE), gray curve and area depict *p* values and highlight frequency range where significant differences (*p* < 0.05, Bonferroni corrected) were detected. Significant differences between female and male groups were obtained in the 35–43 Hz range for PLIs (z_min_ = −4.03, z_max_ = −3.32, corrected *p* < 0.05) and in the 35–46 Hz range for ERSP (z_min_ = −3.41, z_max_ = −3.18, corrected *p* < 0.05). In IGF plots, white circle represents the mean. No differences were found between the groups in IGF_PLI_ (z = −0.53, *p* = 0.59) and IGF_ERSP_ (z = −1.12, *p* = 0.26). Between group comparisons were performed using Wilcoxon rank-sum test.

The phase-locking index (PLI) and event-related spectral perturbation (ERSP) frequency curves for all channels are plotted in Figures 2A and 2B, clearly demonstrating more expressed responses over fronto-central (FC) region, centered on midline electrodes, and overall more pronounced activity in male group. The correlation plots of IGFs extracted at individual channels from PLI (Figure 2C) and ERSP (Figure 2D) measures indicate predominantly positive correlations between electrodes that showed the most pronounced EFR (Figures 2A and 2B). The following patterns of higher correlations can be observed: (1) within and between adjacent ROIs in the fronto-central area, clustered around the midline electrodes; (2) within and between adjacent ROIs in the parieto-occipital area; (3) among fronto-central and parieto-occipital ROIs. Median (inter-quartile range) strength of correlations in the midline fronto-central area (F1, Fz, F2, FC1, FCz, FC2, C1, Cz, and C2) was 0.38 (0.21) for IGF_PLI_ and 0.41 (0.36) for IGF_ERSP_ in female group, and 0.43 (0.20) and 0.38 (0.31) for IGF_PLI_ and IGF_ERSP_ in the male group, respectively.

PLI and ERSP values averaged over midline fronto-central channels (F1, Fz, F2, FC1, FCz, FC2, C1, Cz, and C2) show a clear difference in response to chirp-like stimulus between groups (Figures 2E and 2F). Significant differences between female and male groups were obtained in the 35–43 Hz range for PLIs (z_min_ = −4.03, z_max_ = −3.32, corrected *p* < 0.05) whereas for ERSPs the difference was observed in the 35–46 Hz range (z_min_ = −3.41, z_max_ = −3.18, corrected *p* < 0.05); for both measures, stronger response was observed in males (Figures 2G and 2H). PLI and ERSP averages were computed over the frequencies which showed significant differences between the groups: average PLI was 3.87 (SE = 0.23) in females and 5.34 (SE = 0.27) in males; average ERSP was 1.19 (SE = 0.03) in females and 1.35 (SE = 0.04) in males. For completeness, raw (non-normalized) PLI output is presented in supplementary materials (Figure S1). The topographic representation of the activity in frequency ranges showing significant female/male difference is presented in Figures 2E and 2F. A clear fronto-central distribution of the response is evident, in line with earlier studies utilizing both chirp-like^28^ and single-frequency steady stimulation.^18^

IGFs extracted from PLIs were significantly lower than those obtained from ERSPs in both females (z = −2.01, *p* = 0.04) and males (z = −2.49, *p* = 0.01). In females, mean IGF_PLI_ was 37.55 Hz (SE = 0.88; 30–58 Hz) and IGF_ERSP_ was 39.71 Hz (SE = 1.20; 30–58 Hz). In males, mean IGF_PLI_ was 37.18 Hz (SE = 0.62; 30–44 Hz) and IGF_ERSP_ was 41.08 Hz (SE = 1.15; 30–58 Hz). However, no differences were found between the groups in IGFs: IGF_PLI_ (z = −0.53, *p* = 0.59) and IGF_ERSP_ (z = −1.12, *p* = 0.26) (Figures 2G and 2H).

To note, PLI estimates at IGF and at 40 Hz were positively correlated in both females (rho = 0.72, *p* < 0.01) and males (rho = 0.86, *p* < 0.01); likewise, a strong correlation between values of ERSP at IGF and at 40 Hz was observed in females (rho = 0.89, *p* < 0.01) and males (rho = 0.96, *p* < 0.01). Laterality indices (LIs) revealed no hemispheric differences in either group or measure, with mean LIs being close to zero. At IGF, LI_PLI_ was −0.04 (SE = 0.02) and LI_ERSP_ was −0.004 (SE = 0.008) in females; LI_PLI_ was −0.003 (SE = 0.03) and LI_ERSP_ was 0.009 (SE = 0.01) in males. Similarly, at 40 Hz, females had LI_PLI_ of −0.04 (SE = 0.03) and LI_ERSP_ of −0.02 (SE = 0.006); males had LI_PLI_ of −0.03 (SE = 0.03) and LI_ERSP_ of 0.003 (SE = 0.009). No significant differences in LIs were found between sexes (*p* > 0.14).

## Discussion

In this study we sought to evaluate the sex-related difference in the individual ability to generate gamma-range oscillatory brain activity. To do so, we measured responses to click trains presented in a logarithmic sequence, both in descending and ascending order, covering the 30–60 Hz frequency range.

We analyzed phase-locking index (PLI) and event-related spectral perturbation (ERSP) at each stimulation point and estimated individual gamma frequencies (IGFs), defined as the frequencies eliciting the strongest and most synchronized responses. IGF estimates did not differ between sexes, on average peaking around 37 Hz for PLIs and 40 Hz for ERSPs, spanning 30–58 Hz range and aligning with previous findings using both similar^29^ and alternative methodologies.^39^^,^^41^ We could not find any reports comparing auditory IGFs between sexes in the literature, therefore this aspect cannot be properly discussed. However, both PLIs and ERSPs were significantly higher in males, not only at IGF but across a broader frequency range centered around 35–45 Hz. To align with existing 40-Hz ASSR literature, we extracted PLIs and ERSPs at 40 Hz, which were highly correlated with the corresponding parameters at IGF, reinforcing our previous observations.^28^^,^^29^ These findings support the suggestion by Gransier et al.^43^ that ASSRs within the 30–60 Hz range originate from the same neural generators, with stimulation frequency choice having little impact on relative measures, despite individual variations in peak response frequencies. This also falls in line with our previous observations in clinical samples,^30^^,^^31^ where significant associations with clinical symptoms were observed not for a single frequency but for a range of frequencies around 40 Hz. Although it is not fully clear if EFRs and 40-Hz ASSRs reflect the same network properties, previous studies utilizing both responses reported similar outcomes,^31^^,^^44^ hinting to the potential generalizability of the findings across response types. Finally, exploratory analysis on the asymmetry revealed no hemispheric dominance in EFRs in both males and females, supporting our earlier findings on 40-Hz ASSR.^18^ To note, responses were more prominent in fronto-central area, again aligning with 40-Hz ASSR findings.^18^ Moreover, IGF estimates showed prominent consistency in the fronto-central regions.

Our finding that males exhibit stronger 40-Hz EFRs compared to females aligns with some prior studies highlighting sex-related differences in synchronization to external gamma-range stimulation. A recent study^19^ demonstrated that 40-Hz ASSRs are more synchronized in male mice as compared to female mice. Similarly, stronger phase-locking and response strength was reported in males, but only among left-handed individuals,^18^ suggesting a presence of complex interaction between sex, handedness, and neural synchronization. Additionally, Zakaria et al.^17^ found that females have lower ASSR thresholds (i.e., the lowest sound intensity at which reliable ASSR is observed) at 40 Hz in response to amplitude-modulated 500-Hz carrier sounds and hypothetically linked it to the modulatory role of estrogen on inhibitory action. Indeed, changes of ASSR with fluctuation of estrogen levels were reported in both humans^21^ and mice.^22^ Although our design did not directly assess hormonal influences, hormonal backgrounds inherently differ between sexes and may contribute to interindividual variability in neural responses.^45^^,^^46^ Precise delineation of such influences would require studies combining electrophysiological measures with direct hormonal assessments and longitudinal sampling. In the current study, we enrolled females during the early follicular phase (menses), to capture the state of low sex-hormone levels (both estrogen and progesterone) and to reduce its potential confounding effect.

Studies on schizophrenia,^8^ bipolar disorder,^6^ and autism^47^^,^^48^ frequently report reduced 40-Hz ASSRs. However, the assessment of sex-related effects in the context of neuropsychiatric conditions is extremely rare—we were able to identify only two studies directly evaluating the impact of sex on ASSRs. In a sample of teenage non-psychotic subjects with 22q11.2 deletion syndrome and healthy controls, no effects of sex were found on power and phase-locking of ASSR.^49^ On the contrary, a significant difference between sexes in the combined sample of controls and patients with schizophrenia, with female subjects producing larger gamma amplitudes compared to males,^20^ i.e., contradicting our observation. However, no significant effect of sex was observed on gamma phase-locking. Additionally, two studies investigated ASSR pattern development in animal models of autism separately for male and female animals. Port et al.^50^ demonstrated that male and female mice may have different neural mechanisms governing gamma oscillations and their modulation by inhibitory neurotransmitters. To note, reduced gamma-range ASSR was observed in mice model of autism in males, but not females, when compared to wild type (WT) mice. A considerably lower ASSR can be seen in the group of WT mice when compared to male mice across different stimulation frequencies in Figure 6 of their study^50^; however, no separate statistical evaluation of sex differences in ASSR were carried out in the WT. A recent study^51^ found a difference in developmental trajectory of 40-Hz ASSRs between sexes in the auditory cortex of autism mice model, with males showing impaired ASSR through all developmental stages and females exhibiting intact ASSR at postnatal day 30; however, no developmental sex differences in ASSR were observed in the control animals. These findings collectively indicate that while sex-related effects on 40-Hz ASSRs are rarely examined, available evidence points to potential differences in the underlying neural mechanisms of gamma synchronization. From a physiological standpoint, auditory stimulation in the gamma range not only probes the brain’s capacity for neural entrainment but also reflects cortical excitability,^52^ particularly dependent on parvalbumin-positive (PV^+^) interneuron function.^53^ Given that sex hormones can modulate PV^+^ interneuron activity and inhibitory tone,^54^ sex-related differences in gamma responses may partly arise from divergent excitation-inhibition balance mechanisms rather than from oscillatory coupling per se.

Methodological differences may partly explain the variability in observed sex-related effects on ASSRs. Importantly, sample characteristics should be taken into consideration. In Kirihara et al.,^20^ a considerably lower number of females was recruited in the schizophrenia group. The ratio between healthy controls and patients with schizophrenia in female sample was 1.8, while in male sample it equaled 0.5, although the number of females and males in the control group was identical. This sampling imbalance could have contributed to females showing higher ASSR amplitudes when compared to males given that reduced ASSR is generally reported in schizophrenia.^8^ In other human studies, the difference in the total number of female and male subjects (14/31 in ^50^) and/or relatively small total sample sizes (45 in ^50^; 44 in ^18^) may have underpowered the detection of differences in ASSR between sexes overall^49^ or among the right-handers.^18^ This assumption is also supported by a generally high inter-subject variability of responses to gamma-range stimulation.^28^^,^^29^ In addition, studies assessing sex differences in ASSR did not control for female menstrual cycle,^17^^,^^18^^,^^20^^,^^49^ potentially leading to increased variability in ASSR due to hormonal influence.^21^^,^^22^ Finally, Zakaria et al.^17^ assessed ASSR threshold, which essentially differs from the typical amplitude, power or phase-locking measures. Although lower ASSR thresholds were reported for females, meaning that females could produce synchronized responses at lower stimulus intensities, it is unknown whether lower thresholds would be related to stronger ASSR at higher intensities.

The observed sex-related differences in gamma-range responses are consistent with established neuroanatomical and neurophysiological findings. It has been reported that males generally have a higher volume, thickness, and cortical folding in posterior and temporal cortical regions,^36^ with the latter encompassing primary auditory cortex, the main generator of ASSR.^55^ Furthermore, 40-Hz ASSR was shown to be positively correlated with the cortical thickness.^56^ Additionally, differences in the speed of neuronal communication may further contribute to the sex-related variation in 40-Hz responses. Males often exhibit faster cortical nerve conduction velocities,^37^ likely due to increased myelination and larger axonal diameters,^57^ which facilitate a more efficient signal transmission across cortical networks.

These findings have significant implications for understanding sex-specific periodic response patterns and their relevance to neuropsychiatric conditions characterized by gamma-band dysfunction. Our results of stronger gamma-range response in males emphasize the importance of considering sex as a key variable in ASSR/EFR research and its application in clinical and translational neuroscience.

### Limitations of the study

Although the present study evaluated EFRs to sweep stimuli covering a range of frequencies, it is unclear whether the results of the current study directly translate to classical single-frequency ASSRs. This could be evaluated in future studies by using both types of stimuli. In addition, the present work could not directly assess the relationship between EFRs and hormonal levels due to the lack of hormonal measurements.

## Resource availability

### Lead contact

Requests for further information and resources should be directed to and will be fulfilled by the lead contact, Inga Griškova-Bulanova (inga.griskova-bulanova@gf.vu.lt).

### Materials availability

This study did not generate new materials.

### Data and code availability

• All data reported in this paper will be shared by the lead contact upon request.
• This paper does not report original code. For EEG data processing and analysis, freely available and documented Matlab-based packages were used: – Fieldtrip (https://www.fieldtriptoolbox.org/) and EEGLab (https://sccn.ucsd.edu/eeglab/).
• Any additional information required to reanalyze the data reported in this paper is available from the lead contact upon request.

## Acknowledgments

We thank Dovilė Šimkutė and Povilas Tarailis for help with data collection. This study was supported by the 10.13039/501100004504Research Council of Lithuania (LMTLT agreement no. S-LJB-20-1).

## Author contributions

Conceptualization, I.G.-B.; methodology, A.M. and I.G.B.; formal analysis, A.M.; visualization, A.M.; writing – original draft, A.M. and I.G.-B.; writing – review and editing, A.M. and I.G.-B.; funding acquisition, I.G.-B.

## Declaration of interests

The authors declare no competing interests.

## STAR★Methods

### Key resources table

**Table:** 

REAGENT or RESOURCE	SOURCE	IDENTIFIER
**Deposited data**		

Raw and preprocessed EEG data	Mockevicius et al.^33^	–
Demographic data	Mockevicius et al.^33^	–
Analyzed time-frequency data	This study	–

**Software and algorithms**		

MATLAB 2020a	MathWorks	https://www.mathworks.com/
EEGLab	Delorme & Makeig^58^	https://sccn.ucsd.edu/eeglab/
FieldTrip	Oostenveld et al.^59^	https://www.fieldtriptoolbox.org/

**Other**		

Electroencephalography	ANT Neuro	https://www.ant-neuro.com/
Earphones	Shure	https://www.shure.com/

### Experimental and study participant details

Eighty volunteers (42 females, 2 left-handed; 26.07 ± 4.28 years) comprised the study sample. Characteristics of each participant are provided in Supplementary materials. The data from this sample was used in a previous study (for full details, see^33^). Subjects reported good general health, no history of neurological or psychiatric disorders and no use of any medication. They were asked to fill in Beck Depression Inventory, Second Edition (BDI),^60^ and Beck Anxiety Inventory (BAI)^61^ to evaluate the level of experienced depression and anxiety. To rule out the influence of fluctuating sex hormones, females were asked to participate during the early follicular phase (menses). Subjects were asked to refrain from caffeine consumption and smoking 2 hours before the experiment. The study was approved by the Vilnius Regional Biomedical Research Ethics Committee (no. 2020/3-1213-701), and subjects provided written informed consent.

### Method details

#### EEG data

The data included 64-channel EEG recordings during which participants underwent auditory stimulation using chirp-like sounds consisting of 1.5 ms logarithmically-spaced white noise clicks covering 30-60 Hz frequency range (Figure 1A). The sound pressure level was set at 60 dB. Stimuli included both decreasing (chirp-down) and increasing (chirp-up) parts and lasted ∼1400 ms. 200 repetitions were presented with a variable inter stimulus interval of 700–1000 ms.

The data were preprocessed in Matlab 2020a environment using EEGLab plugin.^58^ Power-line noise was removed using multi-tapering and Thomas F-statistics. The data were visually inspected, and channels with substantial noise (shift, movements) were removed (N = 6.51±4.64). Average reference was applied. Further, an independent component analysis (ICA) was performed and components related to eye movements and cardiac activity were removed (3 components per subject). The removed channels were then reconstructed using a 3D spherical spline method. Continuous data were epoched at -1 to 2 s relative to stimulus onset. Epochs containing noise or artifacts were manually rejected (N = 12.27±12.30).

### Quantification and statistical analysis

Time-frequency analysis was carried out using the Fieldtrip toolbox^59^ in the Matlab 2020a environment. Complex Morlet wavelet decomposition (14 cycles) was performed in 30-60 Hz frequency range and the time window from -1000 to 2000 ms for each epoch. From the obtained complex matrix, phase and amplitude information was extracted to compute phase locking-index (PLI) and event-related spectral perturbation (ERSP), respectively. PLI represents the phase alignment among trials and is expressed as follows:

$$PLI\left(f,t\right)=\frac{1}{N}\left|\sum_{n=1}^{N}e^{i\varphi_{n}\left(f,t\right)}\right|$$

where *N* is the number of trials and *φ*_*n*_*(f,t)* is the phase angle of a single frequency and time point within a current trial.

Event-related spectral perturbation (ERSP) corresponds to power averaged over trials, calculated according to the formula:

$$ERSP\left(f,t\right)=\frac{1}{N}\sum_{n=1}^{N}{\left|X_{n}\left(f,t\right)\right|}^{2}$$

where *X*_*n*_*(f,t)* is the complex wavelet coefficient for each frequency, time point and trial.

Relative baseline normalization was applied to PLI and ERSP, dividing all values by the average in the time period from -500 to 0 ms.

IGFs were obtained separately from PLI and ERSP measures for each subject. Times for each frequency in the chirp stimulus were estimated by computing the cumulative sum of element-wise division of 1 by each element in the frequency vector (30-60). For the chirp-down part, both the frequency vector and the resulting element-wise division vector were flipped. For the chirp-up part, the last element in the chirp-down time vector was added to each element of the cumulative sum vector (Figure 1B). After estimating the times for each frequency, PLI and ERSP measures were averaged in the time windows of 150-ms starting from the frequency onset in both chirp-up and chirp-down parts (300 ms in total).

For visualization purposes, frequency curves from single channels averaged for female and male groups were clustered to ROIs arranged on the anterior-posterior plane, separated by dashed lines. Frontopolar/Fp (Fp1, Fpz, Fp2); Anterior Frontal/AF: AF7, AF3, AF4, AF8); Frontal/F (F7, F5, F3, F1, Fz, F2, F4, F6, F8); Fronto-central/FC (FT7, FC5, FC3, FC1, FCz, FC2, FC4, FC6, FT8); Central/C (T7, C5, C3, C1, Cz, C2, C4, C6, T8); Centroparietal/CP (TP7, CP5, CP3, CP1, CPz, CP2, CP4, CP6, TP8); Parietal/P: (P7, P5, P3, P1, Pz, P2, P4, P6, P8); Parieto-occipital/PO (PO5, PO3, POz, PO4, PO6); Occipital/O: (PO7, O1, Oz, O2, PO8). For statistical evaluation, measures over nine fronto-central channels (F1, Fz, F2, FC1, FCz, FC2, C1, Cz, C2) were averaged.

The frequencies with the highest obtained average PLI or ERSP were set as IGFs (Figure 1C). Spearman correlation coefficients were calculated for single-electrode-based IGFs extracted from PLI and ERSP to visualize the pattern of IGF behavior over the scalp. For statistical evaluation, IGFs extacted from averaged nine fronto-central channels (F1, Fz, F2, FC1, FCz, FC2, C1, Cz, C2) were used.

Furthermore, we performed an exploratory analysis aiming to investigate PLI/ERSP hemispheric lateralization. Laterality index (LI) was computed at IGF (previously extracted from 9 fronto-central channels) and at 40 Hz for PLI and ERSP measures:

$$LI=\frac{L-R}{L+R}$$

where L represents the average PLI/ERSP value in the left ROI (F1, FC1, C1), while R – in the right ROI (F2, FC2, C2). The output was confined within -1 to 1 range, with negative and positive values indicating right and left-side dominance, respectively.

Statistical analysis was carried out in the Matlab 2020a environment. To evaluate sex differences in the obtained measures, the data were divided into female and male groups. Age, BAI and BDI scores were compared between groups using Wilcoxon Rank Sum test for independent samples and the proportions in education levels (tertiary/secondary) were compared using Chi-Square test of independence. PLI and ERSP values at each frequency (30-60 Hz) were compared between groups by applying Wilcoxon Rank Sum test and the resulting p values were corrected using Bonferroni method. The IGFs extracted from PLI and ERSP measures were compared separately in each group by applying Wilcoxon Signed Rank test for dependent samples. Subsequently, IGFs between groups were compared separately for PLI and ERSP measures using Wilcoxon Rank Sum test. In each group, Spearman correlations were computed between PLI/ERSP at IGF and at 40 Hz. LIs for PLI and ERSP at IGF and 40 Hz were compared between groups using Wilcoxon Rank Sum test, whereas single-channel peak frequencies were used for Spearman correlation analysis.
