# Supplementary material for: The ability to drive cortical networks in the low gamma range differs between sexes
Source: iScience. 2026 Feb 4;29(3):114893. doi: 10.1016/j.isci.2026.114893 (PMC12927311; doi:10.1016/j.isci.2026.114893)
Supplement: Document S1. Figure S1 and Table S1 [file mmc1.pdf]

iScience, Volume *29*

Supplemental information

The ability to drive cortical networks in the low gamma range differs between sexes

Aurimas Mockevičius and Inga Griškova-Bulanova

ID	Age	Sex	Race
1	23	F	Caucasian
2	19	F	Caucasian
3	23	F	Caucasian
4	22	F	Caucasian
5	24	F	Caucasian
6	22	F	Caucasian
7	20	F	Caucasian
8	27	F	Caucasian
9	29	F	Caucasian
10	23	F	Caucasian
11	33	F	Caucasian
12	23	F	Caucasian
13	25	F	Caucasian
14	29	F	Caucasian
15	31	F	Caucasian
16	28	F	Caucasian
17	29	F	Caucasian
18	21	F	Caucasian
19	22	F	Caucasian
20	26	F	Caucasian
21	28	F	Caucasian
22	22	F	Caucasian
23	22	F	Caucasian
24	30	F	Caucasian
25	32	F	Caucasian
26	28	F	Caucasian
27	27	F	Caucasian
28	33	F	Caucasian
29	28	F	Caucasian
30	20	F	Caucasian
31	26	F	Caucasian
32	22	F	Caucasian
33	25	F	Caucasian
34	25	F	Caucasian
35	22	F	Caucasian
36	30	F	Caucasian
37	25	F	Caucasian
38	27	F	Caucasian
39	22	F	Caucasian
40	35	F	Caucasian
41	21	F	Caucasian
42	21	F	Caucasian

ID	Age	Sex	Race
43	28	M	Caucasian
44	27	M	Caucasian
45	28	M	Caucasian
46	21	M	Caucasian
47	22	M	Caucasian
48	26	M	Caucasian
49	24	M	Caucasian
50	23	M	Caucasian
51	35	M	Caucasian
52	27	M	Caucasian
53	25	M	Caucasian
54	24	M	Caucasian
55	20	M	Caucasian
56	33	M	Caucasian
57	29	M	Caucasian
58	26	M	Caucasian
59	30	M	Caucasian
60	35	M	Caucasian
61	26	M	Caucasian
62	35	M	Caucasian
63	31	M	Caucasian
64	21	M	Caucasian
65	22	M	Caucasian
66	25	M	Caucasian
67	31	M	Caucasian
68	31	M	Caucasian
69	25	M	Caucasian
70	27	M	Caucasian
71	32	M	Caucasian
72	23	M	Caucasian
73	27	M	Caucasian
74	32	M	Caucasian
75	24	M	Caucasian
76	33	M	Caucasian
77	29	M	Caucasian
78	24	M	Caucasian
79	21	M	Caucasian
80	23	M	Caucasian

Table S1. Age, sex and race of study participants.

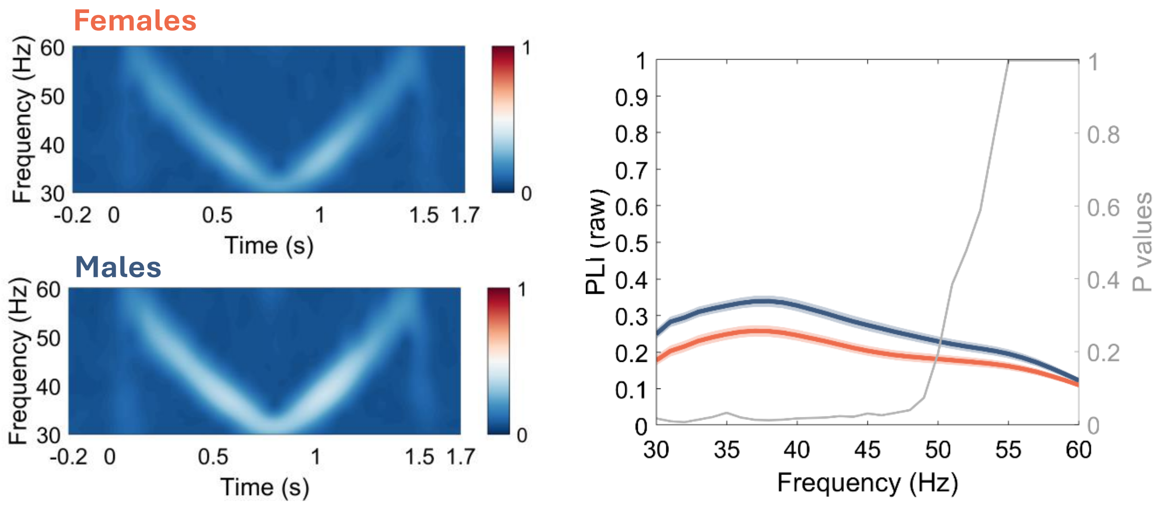

Figure S1. Time-frequency plots of raw (non-normalized) grand-averaged PLI in female and male groups (left) and raw PLI curves with Bonferroni-corrected p values of between-group comparison using Wilcoxon Rank Sum test (right).
